# *Punica granatum* seed oil detracts peritoneal adhesion: Perusing antioxidant, anti‐inflammatory, antifibrotic, and antiangiogenic impacts

**DOI:** 10.14814/phy2.15545

**Published:** 2022-12-21

**Authors:** Hassan Rakhshandeh, Vafa Baradaran Rahimi, Zahra Habibi, Zahra Sirousi, Vahid Reza Askari

**Affiliations:** ^1^ Pharmacological Research Center of Medicinal Plants Mashhad University of Medical Sciences Mashhad Iran; ^2^ Department of Cardiovascular Diseases, Faculty of Medicine Mashhad University of Medical Sciences Mashhad Iran; ^3^ International UNESCO Center for Health‐Related Basic Sciences and Human Nutrition Mashhad University of Medical Sciences Mashhad Iran; ^4^ Applied Biomedical Research Center Mashhad University of Medical Sciences Mashhad Iran

**Keywords:** anti‐inflammatory, antioxidant, peritoneal adhesion, pomegranate seed oil, *Punica granatum*

## Abstract

Peritoneal adhesion is a significant problem following gastrointestinal surgeries, accompanied by a significant economic burden and morbidity for patients. *Punica granatum* seed oil (PSO) possesses antioxidative, anti‐inflammatory, and anticancer effects. Thus, we aimed to evaluate the antiperitoneal adhesive properties of PSO in rats. Forty‐eight Wistar rats (200–250 g) were randomly and equally divided into six groups: sham group, control group; peritoneal adhesion without any treatment, vehicle group; peritoneal adhesion with saline + Tween‐80.5% treatment, and experimental groups; peritoneal adhesion with 0.5%, 1.5%, and 4.5% v/v PSO treatment. In addition, peritoneal adhesion was examined macroscopically along with evaluating the oxidative stress (malondialdehyde [MDA], nitric oxide [NO], and glutathione [GSH]) inflammatory (interleukin [IL]‐6, IL‐1β, and tumor necrosis factor‐α [TNF‐α]), fibrotic (transforming growth factor‐β [TGF‐β]), and angiogenic (vascular endothelial growth factor [VEGF]) factors. Our results revealed that the levels of adhesion scores, MDA, NO, IL‐6, TNF‐α, IL‐1β, TGF‐β, and VEGF, were propagated in the vehicle group while the GSH level was alleviated (*p* < 0.001). In contrast, premedication with PSO, especially at the lowest concentration, notably lessened the levels of adhesion scores, MDA, NO, IL‐6, TNF‐α, IL‐1β, TGF‐β, and VEGF as well as GSH in comparison to the vehicle group following the peritoneal adhesion induction (*p* < 0.001–0.05). As a result, PSO may prevent peritoneal adhesion through its antioxidant, anti‐inflammatory, antifibrotic, and antiangiogenic properties. Therefore, PSO could be considered a beneficial candidate for the treatment of postoperative peritoneal adhesion.

## Graphical abstract

*Punica granatum* seed oil may prevent peritoneal adhesion through its antioxidant, anti‐inflammatory, antifibrotic, and antiangiogenic properties.

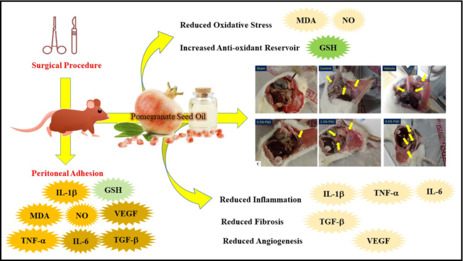

## 1 INTRODUCTION

Peritoneal adhesions are considered an important bothering postsurgical problem that occurs following 63%–97% (Arung et al., 2011; Kössi et al., 2003; Menzies & Ellis, 1990) of abdominal operations and is a great burden for patients and the health system. It may lead to chronic intestinal pain, obstruction and perforation, female infertility, and urologic dysfunctions (Soltany, 2021). However, the severity, time interval to the development of symptoms, and type of clinical complications differ according to the patient's age, number and complexity of surgeries, surgeon skills, and so on (Tang et al., 2020). Generally, three standard strategies have been advised to prevent postoperational adhesions, including physical separation, surgical optimization, pharmacological agents such as glucocorticosteroids, nonsteroidal anti‐inflammatory drugs (NSAIDs), and anticoagulated fibrinolytic agents, inhibitors of the growth factor, and antibiotics. However, the effectiveness of these methods is variable and not assured (Li et al., 2017; Yue et al., 2018).

It has been noticed that the pathophysiology of peritoneal adhesion is associated with fibrinolysis, inflammation, oxidative stress, and angiogenesis (Askari et al., 2018; Ghadiri et al., 2021; Jaafari et al., 2021; Koninckx et al., 2016; Rahimi et al., 2017; Rahmanian‐Devin et al., 2021; Roohbakhsh et al., 2020). Indeed, the increased secretion of signaling molecules such as tumor necrosis factor‐alpha (TNF‐α), interleukin (IL)‐1β, IL‐6, transforming growth factor‐beta (TGF‐β), platelet‐derived growth factor (PDGF), and vascular endothelial growth factor (VEGF) from monocytes, macrophages, fibroblasts, and mesothelial cells in the adhesion site is responsible for adhesion formation (Askari et al., 2018; Baradaran Rahimi & Askari, 2022; Ghadiri et al., 2021; Jaafari et al., 2021; Maciver et al., 2011; Rahimi et al., 2017; Rahmanian‐Devin et al., 2021; Roohbakhsh et al., 2020).

*Punica granatum* (pomegranate), belonging to the Punicaceae, has been extensively used as a traditional medication in different cultures for thousands of years (Baradaran Rahimi et al., 2020). Fruits are the edible part of the pomegranate and contain a tremendous amount of seeds, approximately between 40 and 100 g/kg, which remain a waste after processing (Baradaran Rahimi et al., 2020; Dadashi et al., 2016). Pomegranate seed oil (PSO) comprises 12%–20% of total seed weight, consisting of 8% saturated fatty acids, 10% mono‐unsaturated, 10% di‐unsaturated, and about 70% conjugated acids. It has been reported that cold‐pressed PSO includes punicic acid (65.3%), palmitic acid (4.8%), stearic acid (2.3%), oleic acid (6.3%), linoleic acid (6.6%), and isomers of punicic acid (14.2%) (Lim, 2012). Furthermore, PSO has several pharmacological properties, including antioxidant, anti‐inflammatory, anticancer, antiobesity, antidiabetic, and antihyperlipidemic activities (Baradaran Rahimi et al., 2020; Lansky & Newman, 2007).

Therefore, in this study, we aimed to investigate the anti‐inflammatory, antioxidant, and antiadhesive properties of PSO following postoperative peritoneal adhesion in rats.

## 2 MATERIALS AND METHODS

### 2.1 Chemicals and kits

Ketamine (10%), xylazine (2%), and acepromazine (1%) were prepared by Alfasan Co, Netherlands. Biochemical mediators colorimetric kits consisting of malondialdehyde (MDA), nitric oxide (NO), and glutathione (GSH) were obtained from ZellBio Company. Immunological mediators enzyme‐linked immunosorbent assay (ELISA) kits, including IL‐6, IL‐1β, TNF‐α, TGF‐β, and VEGF, were purchased from Diaclone Co.

### 2.2 Preparation of pomegranate seed oil

Pomegranate fruits were purchased from the local market of Mashhad, Khorasan province, Iran, in December 2018. The seeds were then carefully separated and dried in the shadow and at room temperature. Next, for preparing the oil, the dried seeds were undergone cold pressure using VN‐10330 Cold Press Oil Machine, Niavaran Kohan Asia. Afterward, the obtained oil was filtrated utilizing a polytetrafluoroethylene (PTFE) 0.22 μm syringe filter to remove suspended particles and sterilize the oil. The density of the oil was measured at 25°C equalling 0.81 g/ml.

### 2.3 Animals and ethical statement

Thirty‐six male Wistar rats (200–250 g) were obtained from the animal laboratory of the Faculty of Medicine, Mashhad University of Medical Sciences. Rats were housed in separated standard cages and ventilated rooms with a 12‐h/12‐h natural light/dark cycle at a temperature of 21 ± 2°C and humidity of 60 ± 3%. The animals had free access to tap water and food. All animals received human care in conformity with the institutional instructions of Mashhad University of Medical Sciences (IR.MUMS.MEDICAL.REC.1397.703, 25‐Sep‐2018). The research was prospectively reviewed and approved by a duly constituted ethics committee of Mashhad University of Medical Sciences with the Ethical ID “IR.MUMS.MEDICAL.REC.1397.703” confirmed on 25/8/2018.

### 2.4 Surgical technique

The surgical procedure was accomplished according to our previously published article (Askari et al., 2018; Ghadiri et al., 2021; Jaafari et al., 2021; Rahimi et al., 2017; Rahmanian‐Devin et al., 2021; Roohbakhsh et al., 2020). Briefly, rats were anesthetized using intraperitoneal (i.p.) injection of a mixture of ketamine (100 mg/kg), acepromazine (3 mg/kg), and xylazine (10 mg/kg) (Askari et al., 2018; Ghadiri et al., 2021; Jaafari et al., 2021; Rahimi et al., 2017; Rahmanian‐Devin et al., 2021; Roohbakhsh et al., 2020). After that, an incision measuring 3 cm was carefully made in the abdominal midline in order to gain access to the abdominal cavity. Thereafter, the intra‐abdominal adhesion was generated using soft sterile sandpaper on one side of the cecum. Besides that, the peritoneum was washed thoroughly with 2 ml of the extract or vehicle, and 4–0 polyglactin sutures were used to close the abdominal wall. Noteworthy, the surgeon was only blinded to the treatment groups at the time of the operation, but not to the sham group. The sham group received only the surgery without peritoneal adhesion induction.

After the operation, the rats were given 7 days to recover in the recovery room while they remained in their cages. In addition, to reduce the development of the risk of a wound and operation infection, each rat was administered a single dose of the antibiotic cefazolin (300 mg/kg intramuscularly; i.m.) as soon as the surgical procedure was finished (Askari et al., 2018; Ghadiri et al., 2021; Jaafari et al., 2021; Rahimi et al., 2017; Rahmanian‐Devin et al., 2021; Roohbakhsh et al., 2020).

### 2.5 Investigational groups

A total of 36 rats were randomly split up into six groups of six, each of which is described below:

Group 1: Sham group; received the surgical procedure without peritoneal adhesion induction.

Group 2: Control group; received the surgical procedure with peritoneal adhesion induction without any treatment.

Group 3: Vehicle group; received the surgical procedure with peritoneal adhesion induction with saline + Tween‐80 5% treatment.

Group 4–6: Investigational groups; received the surgical procedure with peritoneal adhesion induction and 0.5, 1.5, or 4.5% v/v PSO + Tween‐80 5% treatment.

After the induction of adhesion, the lavage method involved washing the injured and front sides of the peritoneum with either 2 ml of the vehicle or various concentrations of the extract, depending on the grouping (Askari et al., 2018; Ghadiri et al., 2021; Jaafari et al., 2021; Rahimi et al., 2017; Rahmanian‐Devin et al., 2021; Roohbakhsh et al., 2020).

In this study, we used the body weight—the stratified randomization method. Briefly, according to the experimental design, we split the rats into six groups of six based on body weight. Then, the largest six rats were assigned to block one, the next largest six rats to block two, and continued until the end. Afterward, the rats were allocated from these eight groups to the six experimental groups. There were no differences between groups in the aspect of weight at the first and end of the study.

### 2.6 Macroscopic scaling of adhesion

In order to investigate the adhesion, a laparotomy was carried out 7 days after the surgery, and the peritoneal adhesions were graded blindly by two independent researchers using Nair et al. (1974; Table 1) and adhesion scheme scoring systems (Table 2) (Askari et al., 2018; Ghadiri et al., 2021; Jaafari et al., 2021; Rahimi et al., 2017; Rahmanian‐Devin et al., 2021; Roohbakhsh et al., 2020). In addition, the peritoneal lavage fluid was collected for further evaluation.

**TABLE 1:** Scoring system for peritoneal adhesion according to the Nair et al. criteria (Nair et al., 1974)

Grade	Description of adhesive bands
0	The complete absence of adhesions
1	Only one band of adhesions among the viscera or between one viscera and the abdominal wall
2	Two bands: among viscera or from viscera to abdominal wall
3	More than two bands: among viscera or from viscera to the abdominal wall or all intestine making a mass without adhesion to the abdominal wall
4	Viscera adhered directly to the abdominal wall, independent of the number and the extension of adhesion bands

**TABLE 2:** Scoring system for peritoneal adhesion according to Adhesion Scoring Scheme (Nair et al., 1974; Rahimi et al., 2017; Rahmanian‐Devin et al., 2021)

Grade	Description of adhesive bands
0	Absence of adhesions
1	A thin layer of adhesion
2	More than a thin layer of adhesion
3	Thick adhesive tissue attached to the surgical site
4	Thick adhesive tissue attached to different areas of the abdomen
5	Thick adhesive tissue containing blood vessels or too much adhesive tissue or organ adhesive tissue

### 2.7 Peritoneal lavage fluid preparation

Following the laparotomy performed on the rats, the peritoneal lavage fluid was prepared by using 2.5 ml of sterile phosphate‐buffered saline (PBS). In greater detail, the entire region of the peritoneum was washed twice, and then the collected fluid was centrifuged at a speed of 3000 rpm for 5 min while the temperature was set at 4°C. Finally, the supernatant was separated out for the purpose of further research.

### 2.8 Total protein measurement method

An assay known as the Bradford protein assay was carried out to determine the overall protein concentration in a sample (Askari et al., 2018; Bradford, 1976; Ghadiri et al., 2021; Jaafari et al., 2021; Rahimi et al., 2017; Rahmanian‐Devin et al., 2021; Roohbakhsh et al., 2020). Because of this, the Coomassie Brilliant Blue G‐250 dye, which weighed 10 mg, was first dissolved in 50 ml of ethanol with a concentration of 96%. After that, 10 ml of phosphoric acid with an 85% concentration was added to the solution, and the total volume was brought up to 100 ml. The standard curve was then constructed using a solution of bovine serum albumin at a concentration of 4 mg/ml. After that, 20 μl of the sample were poured into the 96‐well microplate, and then 200 μl of the Bradford reagent were added. After waiting for 5 min, a final reading of the light absorption was taken at 595 nm using a microplate reader.

### 2.9 Assessment of oxidative and antioxidative factors

The levels of MDA and NO, as oxidative stress indexes, as well as GSH, as an antioxidative marker, were measured in the peritoneal lavage fluid using the commercial colorimetrical assay kits (ZellBio Company) according to the manufacturer's guidelines (Askari et al., 2020; Rahimi et al., 2018).

### 2.10 Measurement of inflammatory markers

The levels of inflammatory markers, IL‐6, TNF‐α, and IL‐1β, were determined in the peritoneal lavage fluid using the commercial ELISA kits (Diaclone Company) according to the manufacturer's protocols.

### 2.11 Assessment of fibrosis and angiogenesis biomarkers

The levels of TGF‐β, as a fibrosis marker, as well as VEGF, as an angiogenesis factor, were assessed in the peritoneal lavage fluid using the commercial ELISA kits (Diaclone Company) according to the manufacturer's manuals.

### 2.12 Statistical analysis

Data were analyzed using Graph Pad Prism (version 6.01) software and illustrated as mean ± SEM and median ± range for parametric and nonparametric data, respectively. Parametric data were analyzed using one‐way ANOVA followed by Tukey–Kramer's post hoc test, and the Kruskal–Wallis test analyzed nonparametric results followed by Dunn's multiple comparisons post‐test. Furthermore, *p* values lower than 0.001, 0.01, and 0.05 were considered statistically significant.

## 3 RESULTS

### 3.1 The effect of PSO on peritoneal adhesion scoring

Our result illustrated that all rats underwent surgical processes and interventions and survived until the study's end. Thus, the survival rates for all groups were 100%.

**FIGURE 1:**
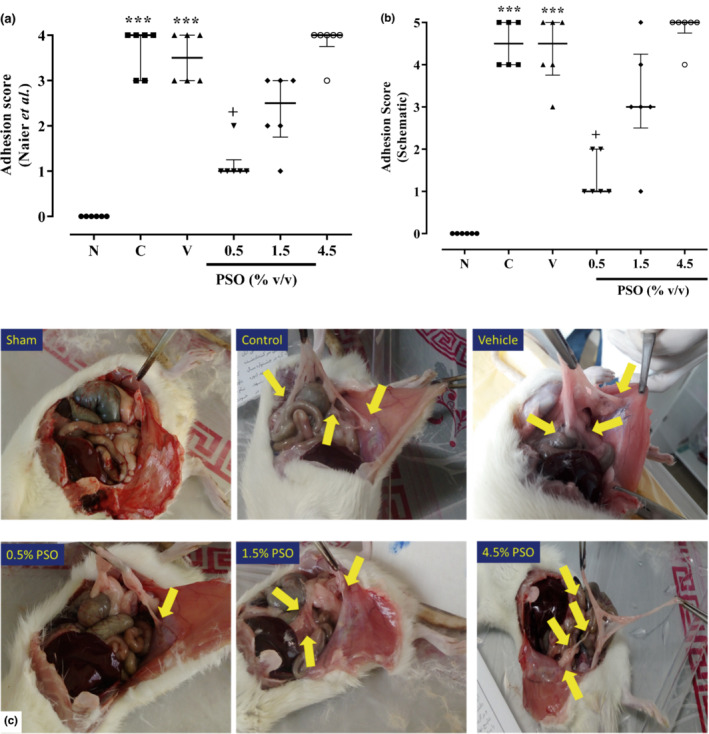
The effect of different concentrations of PSO on adhesion scores following the postoperational‐induced peritoneal adhesion based on (a) Nair and (b) Adhesion Scheme scoring systems, (c) Illustration of adhesion bands in different groups. Data are presented as the median ± range (*n* = 6). Yellow arrows indicate the adhesion bands. ***p < 0.001 compared to the sham group and ^+^ *p* < 0.05 compared to the vehicle group. N: sham, C: control, V: vehicle, PSO: pomegranate seed oil.

According to both the Nair and Adhesion Scheme scoring systems, the adhesion scores were notably more outstanding in both control and vehicle groups than in the sham group (*p* < 0.001 for both cases, Figure 1a,b). However, there was no significant difference between the control and vehicle groups. Howbeit, the adhesion score was markedly decreased following the treatment with PSO (0.5% v/v) in comparison with the vehicle group (*p* < 0.05, Figure 1a,b).

### 3.2 The effect of PSO on oxidative and antioxidative factors

**FIGURE 2:**
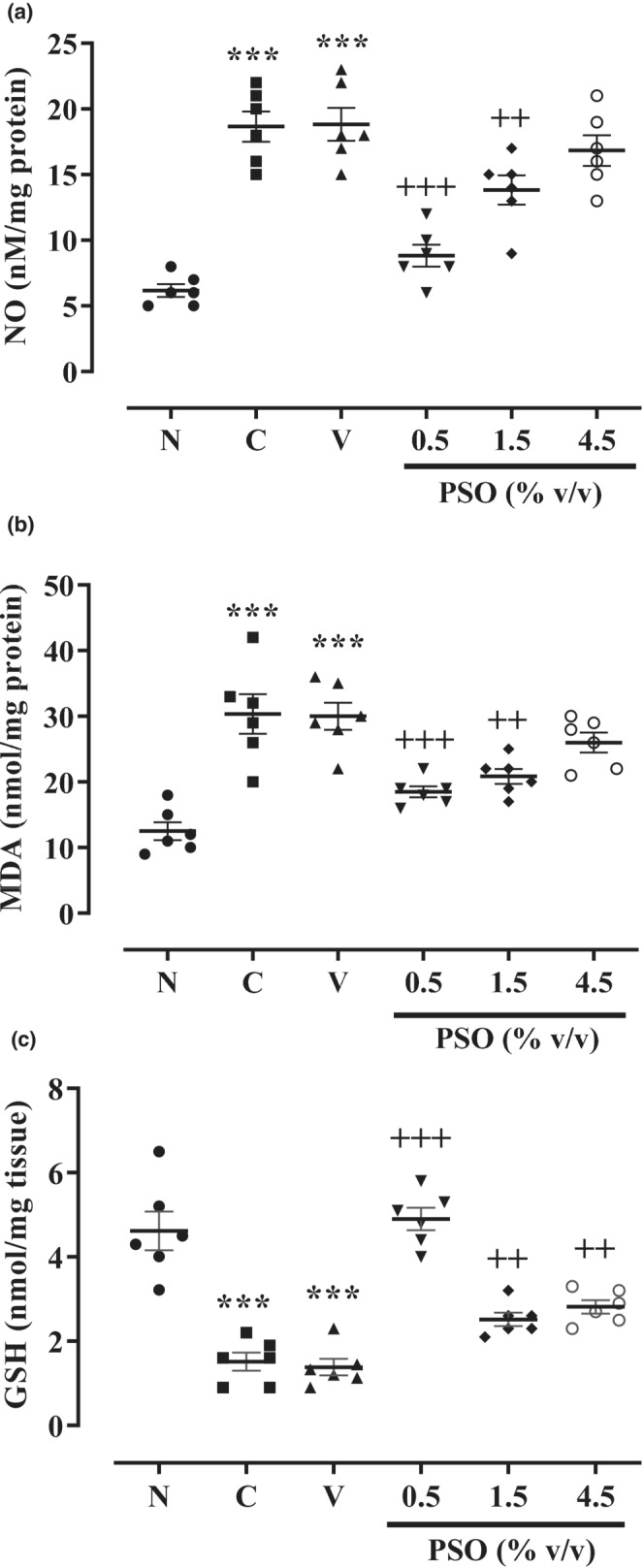
The effect of different concentrations of PSO on the levels of (a) NO, (b) MDA, and (c) GSH following the postoperational‐induced peritoneal adhesion. Data are presented as mean ± SEM (*n* = 6). ***p < 0.001 compared to the sham group and ^+++^ *p* < 0.001 and ^++^ *p* < 0.01 compared to the vehicle group. Abbreviations: N: sham, C: control, V: vehicle, PSO: pomegranate seed oil.

As illustrated in Figure 2, the levels of NO and MDA were meaningfully elevated in both control and vehicle groups compared to the sham group (*p* < 0.001 for both cases, Figure 2a,b). However, no significant differences were observed between the control and vehicle groups. In contrast, the two lower concentrations of PSO (0.5 and 1.5% v/v) remarkably diminished NO (*p* < 0.001 and *p* < 0.01, respectively, Figure 2a) and MDA (*p* < 0.001 and *p* < 0.01, respectively, Figure 2b) levels in comparison with the vehicle group following the peritoneal adhesion induction.

On the other hand, the level of GSH was considerably mitigated in both control and vehicle groups (*p* < 0.001 for both cases, Figure 2c) compared to the sham group. Our results also indicated that there were no differences between the effects of control and vehicle groups on GSH level. Reciprocally, all three concentrations of PSO (0.5, 1.5, and 4.5% v/v) strikingly enhanced the GSH level in comparison with the vehicle group (*p* < 0.001, *p* < 0.01, and *p* < 0.01, respectively, Figure 2c) after the peritoneal adhesion induction.

### 3.3 The effect of PSO on inflammatory biomarkers

**FIGURE 3:**
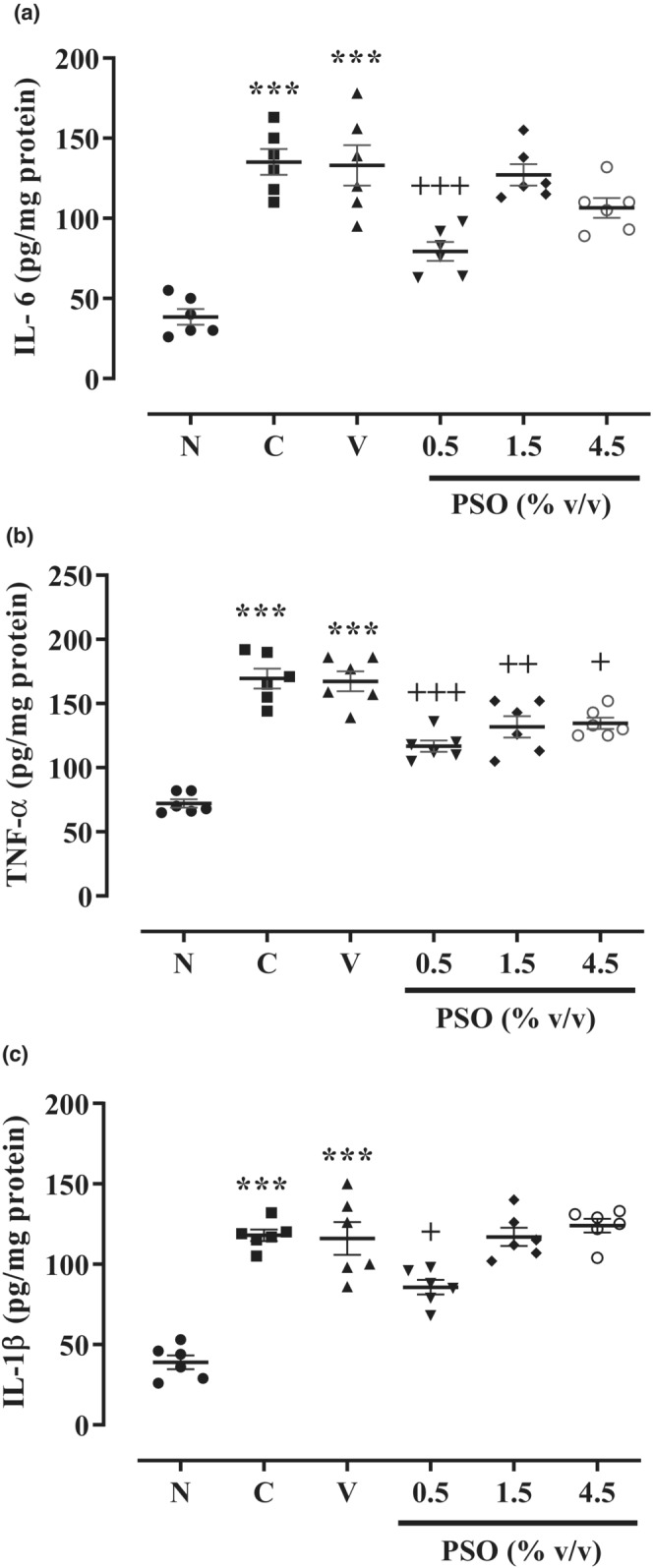
The effect of different concentrations of PSO on the levels of (a) IL‐6, (b) TNF‐α, and (c) IL‐1β following the postoperational‐induced peritoneal adhesion. Data are presented as mean ± SEM (*n* = 6). ***p < 0.001 compared to the sham group and ^+++^ *p* < 0.001, ^++^ *p* < 0.01, and ^+^ *p* < 0.05 compared to the vehicle group. Abbreviations: N: sham, C: control, V: vehicle, PSO: pomegranate seed oil.

Our results revealed that the levels of IL‐6, TNF‐α, and IL‐1β were firmly propagated in both control and vehicle groups compared to the sham group (*p* < 0.001 for all cases, Figure 3a–c). In contrast, only the lowest concentration of PSO (0.5% v/v) notably attenuated the IL‐6 and IL‐1β levels in comparison with the vehicle group (*p* < 0.001 and *p* < 0.05, respectively, Figure 3a–c). In addition, the level of TNF‐α was markedly suppressed following the treatment with all three concentrations of PSO (0.5, 1.5, and 4.5% v/v) compared to the vehicle group (*p* < 0.001, *p* < 0.01, and *p* < 0.05, respectively, Figure 3b).

### 3.4 The effect of PSO fibrosis and angiogenesis markers

**FIGURE 4:**
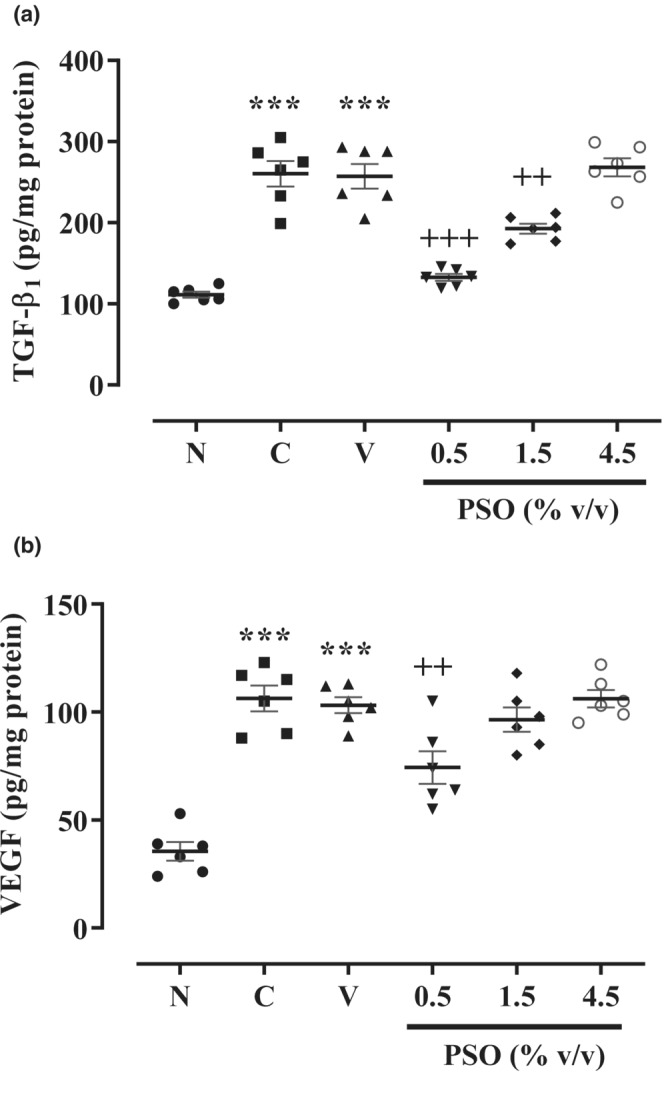
The effect of different concentrations of PSO on the levels of (a) TGF‐β1 and (b) VEGF following the postoperational‐induced peritoneal adhesion. Data are presented as mean ± SEM (*n* = 6). ***p < 0.001 compared to the sham group and ^+++^ *p* < 0.001 and ^++^ *p* < 0.01 compared to the vehicle group. Abbreviations: N: sham, C: control, V: vehicle, PSO: pomegranate seed oil.

As shown in Figure 4a,b, the TGF‐β, as a fibrosis marker, and VEGF, as an angiogenesis marker, were meaningfully elevated in the control and vehicle compared to the sham group (*p* < 0.001 for both cases). Our results also figured out no significant differences between the effects of vehicle and control groups. Reciprocally, the two lower concentrations of PSO (0.5 and 1.5% v/v) remarkably alleviated the TGF‐β level in comparison with the vehicle group (*p* < 0.001 and *p* < 0.01, respectively, Figure 4a). Moreover, only the lowest concentration of PSO (0.5% v/v) considerably prevented the level of VEGF compared to the vehicle group (*p* < 0.01, Figure 4b).

## 4 DISCUSSION

As far as we know, this is the first experiment evaluating the impacts of PSO on the prevention of operational‐induced peritoneal adhesion. This study results revealed that PSO premedication significantly alleviated the adhesion score and the levels of inflammatory cytokines, fibrosis, angiogenesis, and oxidative factors while propagating the antioxidative markers following the postoperational‐induced peritoneal adhesion.

Postoperative peritoneal adhesion is a crucial difficulty in gastrointestinal surgeries and a tremendous economic burden. Moreover, it leads to morbidity and various complications that may present even many years after the surgery (Arung et al., 2011; Askari et al., 2018; Ghadiri et al., 2021; Jaafari et al., 2021; Rahimi et al., 2017; Rahmanian‐Devin et al., 2021; Roohbakhsh et al., 2020). It has been emphasized that multiple factors are associated with developing postsurgical peritoneal adhesion, including hemorrhage, bacterial infections, ischemia, and trauma (Schnüriger et al., 2011). However, no approved treatment has been found yet for all medicines commonly consumed to prevent adhesion, such as anti‐inflammatory, antioxidants, antibacterial, anticoagulants, and fibrinolytic agents (Kamel, 2010).

In this study, according to our previous articles, to induce postoperative peritoneal adhesion, we used the scraping model due to its adhesion to the adhesion formed naturally following abdominopelvic surgeries (Askari et al., 2018; Ghadiri et al., 2021; Jaafari et al., 2021; Rahimi et al., 2017, 2017; Rahmanian‐Devin et al., 2021; Roohbakhsh et al., 2020). Additionally, the Nair scoring system was used for the macroscopic evaluation of adhesion, which scores adhesion from zero to four grades (Askari et al., 2018; Ghadiri et al., 2021; Jaafari et al., 2021; Nair et al., 1974; Rahimi et al., 2017; Rahmanian‐Devin et al., 2021; Roohbakhsh et al., 2020). Our results suggested that the control group significantly increased the adhesion score. At the same time, treatment with PSO (0.5, 1.5, and 4.5% v/v) concentration dependently decreased the adhesion score following the postoperative‐induced peritoneal adhesion based on both Nair and Adhesion Scheme scoring systems. Similarly, in our previous study, the control group provided a significant increment in adhesion score, while treatment with *Rosmarinus officinalis* extract (1, 2, and 4% v/v) provided a substantial decrement in adhesion score following the scraping model‐induced peritoneal adhesion (Roohbakhsh et al., 2020). Moreover, it has been demonstrated that the control treatment enhanced the adhesion grade, while it was diminished by garlic oil (5 ml/kg) treatment following the peritoneal adhesion induced by the scraping model (Sahbaz et al., 2014). These studies are in line with our results and could support our findings.

Interestingly, our previous study found that pomegranate peel extract (PPEx) could reduce peritoneal adhesion by alleviating adhesion formation, IL‐6, TNF‐α, TGF‐β1, VEGF, NO, and MDA, and stimulating antioxidative factors (Ghadiri et al., 2021). This similar effect can be described by the secondary metabolites found in both the peel and seed of pomegranate, such as ellagic acid (Ghadiri et al., 2021). In addition, it has been noted that ellagic acid and ellagitannins have beneficial and protective effects against different disorders (Baradaran Rahimi et al., 2020; Ghadiri et al., 2021; Rahimi et al., 2018).

It has been emphasized that oxidative stress is an increasingly important factor in angiogenesis and fibrosis by stimulating fibroblast cell proliferation in the injured area (Gao et al., 2017). Conversely, antioxidant agents may prevent postsurgical‐induced peritoneal adhesion. In this case, Corrales et al. reported that i.p. administration of vitamin E diminished the postoperative adhesion similar to the carboxymethyl‐cellulose membrane (Corrales et al., 2008). Therefore, we measured the levels of MDA and NO as oxidative markers and GSH as an antioxidative factor after the postoperational peritoneal adhesion. This study demonstrated that PSO (0.5, 1.5, and 4.5% v/v) meaningfully mitigated MDA and NO levels while enhancing GSH content following the postoperational‐induced peritoneal adhesion. Following our results, Yayla and co‐workers figured out that PSO (0.32 and 0.64 ml/kg, i.p.) attenuated the MDA and TNF‐α levels as well as NADPH oxidase activity while augmented superoxide dismutase (SOD) activity and GSH level following the ovarian ischemia/reperfusion injury in rats (Yayla et al., 2018). Similarly, a nano‐droplet formulation of pomegranate seed oil suppressed MDA and oxidative stress levels in brain tissue of experimental autoimmune encephalomyelitis (EAE) mice as an established model of multiple sclerosis (MS) (Binyamin et al., 2015). Al‐Sabahi et al. noticed that PSO (40 μg) provided a significant decrement in lactate dehydrogenase, reactive oxygen species (ROS), and MDA levels while promoting GSH level and SOD and glutathione peroxidase (GPx) activities in 3‐nitropropionic acid‐(3‐NP)‐induced cytotoxicity in rat pheochromocytoma PC12 neuronal cells (Al‐Sabahi et al., 2017). In addition, PSO (25 μg/mL) alleviated the NO and TNF‐α levels as well as inducible nitric oxide synthase (iNOS) expression in lipopolysaccharide (LPS)‐stimulated BV‐2 microglia cell (Račková et al., 2014). Saha and co‐workers showed that punicic acid (0.5% of total lipid given; orally), which comprise 70%–80% of PSO, significantly increased SOD, CAT, and GPx activities as well as GSH level while decreased NOS activity in serum, liver, and brain tissue of rats following the oxidative stress generated by sodium arsenite (Saha & Ghosh, 2009). The results of these studies are in line with our results regarding the antioxidative effects of PSO.

It has been reported that the levels of inflammatory cytokines, including IL‐1β, IL‐6, and TNF‐α were elevated in serum and peritoneal fluid of patients undergoing peritoneal surgeries, which indicates the critical role of inflammation and inflammatory cytokines in the development of adhesion (Soltany, 2021). Furthermore, the postoperative levels of inflammatory cytokines such as TNF‐α and IL‐1β in serum and peritoneal fluid are directly related to adhesion formation's severity (Tang et al., 2020). Therefore, in this study, we determined the effects of PSO on IL‐1β, IL‐6, and TNF‐α levels following the postoperational‐induced peritoneal adhesion. We showed that the lowest concentration of PSO (0.5% v/v) diminished IL‐6 and IL‐1β levels, and all three concentrations of PSO (0.5%, 1.5%, and 4.5% v/v) inhibited the TNF‐α level. In line with our results, Harzallah et al. supported that PSO (2 ml/kg/day; orally) reduced the plasma levels of IL‐6 and TNF‐α while enhancing the levels of IL‐10 anti‐inflammatory cytokine in high‐fat and high‐sucrose diet‐induced obese mice (Harzallah et al., 2016). In addition, PSO (1.5%, orally) decreased the gene expression levels of IL‐6, IL‐8, IL‐12, IL‐23, and TNF‐α in the ileum of the necrotizing enterocolitis rat model (Coursodon‐Boyiddle et al., 2012). Boussetta and co‐workers demonstrated that PSO (2% v/w; orally) ameliorated 2, 4, 6‐trinitrobenzene sulfonic acid (TNBS)‐induced colon inflammation in rats (Boussetta et al., 2009). Moreover, punicic acid (5, 10, and 30 μM) significantly attenuated the levels of IL‐6, IL‐1β, and IFN‐γ following the TNF‐α‐induced insulin resistance in three T3‐L1 adipocyte cells (Anusree et al., 2018). These studies may confirm our results regarding the anti‐inflammatory properties of PSO.

Numerous human and animal studies emphasized the importance of TGF‐β in the development of postoperative adhesion. In addition, the administration of directed neutralizing antibodies against TGF‐β significantly alleviated the incidence of adhesion formation (Chegini, 2008). Moreover, VEGF is another crucial marker of adhesion formation, while sunitinib (40 mg/kg in 100 L methylcellulose; orally), an antagonist of VEGF receptor‐2 (Kim et al., 2008), as well as bevacizumab (2.5 mg/kg; i.p.), a monoclonal antibody against VEGF, prevent the postsurgical peritoneal adhesion in a mice model (Basbug et al., 2011). Interestingly, our results revealed that PSO notably mitigated TGF‐β and VEGF levels following the postoperational‐induced peritoneal adhesion. Consistent with our results, Toi et al. suggested that PSO (100–200 μg/mL) provided a significant decrement in VEGF levels in estrogen‐sensitive (MCF‐7) or estrogen‐resistant (MDA‐MB‐231) human breast cancer cells (Toi et al., 2003). Similarly, a hydrophilic fraction of PSO (0.12–0.6 μl) decreased the levels of VEGF, IL‐2, IL‐6, IL‐12, and TNF‐α in MCF‐7 and MDA‐MB‐231 cells (Costantini et al., 2014). Wei and co‐workers revealed that 50% hydro‐ethanolic extract of pomegranate seed (100 mg/kg, orally) diminished the TGF‐β1 levels in rats' liver fibrosis induced by carbon tetrachloride (Wei et al., 2015). In addition, Mete et al. reported that punicic acid (9.85 μl/mL) significantly reduced the expression levels of VEGF and TGF‐β1 in the T98 glioblastoma cell line (Mete et al., 2019). The results of these studies are in line with our results regarding the antiangiogenic and antifibrotic effects of PSO.

## 5 CONCLUSION

This study suggested that PSO may improve the postoperational‐induced peritoneal adhesion by alleviating the adhesion score, oxidative stress, inflammatory, fibrosis, and angiogenesis markers while increasing antioxidative factors. Therefore, PSO may be a promising candidate for preventing and treating postsurgical peritoneal adhesions.

## AUTHOR CONTRIBUTIONS

VRA, VBR, and HR proposed the conception; VRA and HR designed and implemented the experiments; ZH, HR, ZS, VBR, and VRA participated in animal assays; VRA and VBR participated in the data processing and statistical analysis; VBR and VRA wrote and revised the article. All authors read and approved the final manuscript.

## CONFLICT OF INTEREST

The authors declare that they have no conflict of interest to disclose.

## ETHICAL STATEMENT

The research was prospectively reviewed and approved by a duly constituted ethics committee of Mashhad University of Medical Sciences with the Ethical ID “IR.MUMS.MEDICAL.REC.1397.703” confirmed on 25/8/2018.

## CONSENT FOR PUBLICATION

All authors read the manuscript and agreed to publish it.
